# Supplementary material for: High Stretch Modulates cAMP/ATP Level in Association with Purine Metabolism via miRNA–mRNA Interactions in Cultured Human Airway Smooth Muscle Cells
Source: Cells. 2024 Jan 5;13(2):110. doi: 10.3390/cells13020110 (PMC10813996; doi:10.3390/cells13020110)
Supplement: Supplementary file 1 [file cells-13-00110-s001.zip › Table S1 new.pdf]

Table S1. Primer sequence of mRNA ands miRNAs.

No.	Genes	Forward primer	Reverse primer
1	AK4	GCCCAGGCTAATCTATGAAG	CAAGGAGCTCAAAAGCCTAT
2	AK7	GACTTTGCGGTGGAGACGTA	CACTGAGTGCAGAGACTGCC
3	ENPP1	GGTGGACTTCTTCCTGTTATTA	GGTGACAATGCTGTAGTGA
4	PDE4D	GACCAATGTCTCAGATCAGTGG	GTCAAGGGCCGGTTACCAG
5	PDE7B	AAGGCTGCTTCGTGGAATTA	TCCATTTGTCAAGCGATCAA
6	GADPH	TGACGCTGGGGCTGGCATTG	GGCTGGTGGTCCAGGGGTCT
7	β-actin	GGATGCAGAAGGAGATCACTG	CGATCCACACGGAGTACTTG
8	miR-370-5p mimics	CAGGUCACGUCUCUGCAGUUAC	GUAACUGCAGAGACGUGACCUG
9	Mimics NC	UUCUCC GAACGUGUCACGUUU	ACGUGACACGUUCGGAGAAUU
10	miR-370-5p inhibitor	GUAACUGCAGAGACGUGACCUG	
11	Inhibitor NC	CAGUACUUUUGUGUAGUACAA	
