# Supplementary material for: High Stretch Modulates cAMP/ATP Level in Association with Purine Metabolism via miRNA–mRNA Interactions in Cultured Human Airway Smooth Muscle Cells
Source: Cells. 2024 Jan 5;13(2):110. doi: 10.3390/cells13020110 (PMC10813996; doi:10.3390/cells13020110)
Supplement: Supplementary file 1 [file cells-13-00110-s001.zip › Table S2.pdf]

Table S2. 283 targ DE-mRNAs by 12 DE-miRNAs.

Genes	FC	P_{adjust}	DE-miRNAs
IL1A	45.345	5.502861296	miR-543
F2RL3	24.101	4.591025475	miR-485-3p
BHLHA15	17.985	4.168720779	miR-370-5p
HRK	17.273	4.11043176	miR-485-3p
TMCC3	16.224	4.020096691	miR-485-3p
RRAGD	15.554	3.95920424	miR-543
HYOU1	14.05	3.812462457	miR-485-3p
SLC3A2	13.999	3.807271338	miR-27b-5p
IFI30	13.957	3.80293007	miR-27b-5p
KLF15	13.286	3.731836449	miR-485-3p
PRDM16	12.153	3.603278416	miR-543
ATF3	12.089	3.595582725	miR-485-3p
KCP	8.573	3.099880343	miR-370-5p
REPS2	7.85	2.972600877	miR-543
IGSF3	7.468	2.900758057	miR-543
YJEFN3	6.691	2.742189302	miR-485-3p
FLRT2	6.636	2.730323526	miR-370-5p
SEL1L	6.585	2.719090373	miR-485-3p
PTPRR	6.466	2.692924138	miR-485-3p
RAB3IP	6.032	2.592603832	miR-543
BRCA2	5.981	2.580366517	miR-27b-5p
MCTP1	5.836	2.544978935	miR-370-5p
TGM1	5.817	2.540227543	miR-335-3p
SPOCK2	5.746	2.522532978	miR-485-3p
RENBP	5.686	2.507343735	miR-370-5p
OCLN	5.343	2.417728628	miR-543
LTB4R	5.2	2.37855433	miR-335-3p
SLC16A6	5.169	2.369769434	miR-543
EPHB1	5.157	2.366633083	miR-370-5p
MKNK2	4.775	2.255592335	miR-27b-5p
TLR2	4.705	2.234105732	miR-27b-5p
PLCG2	4.693	2.23058079	miR-27b-5p
SLC39A8	4.677	2.225604049	miR-370-5p
KDR	4.589	2.198080019	miR-485-3p
AK7	4.575	2.193814878	miR-370-5p
KYNU	4.538	2.182061652	miR-27b-5p
LRATD2	4.419	2.143591382	miR-485-3p
FAM174B	4.382	2.131666277	miR-370-5p
HSPA13	4.315	2.109268781	miR-543

CNNM2	4.296	2.102989149	miR-543
DMKN	4.146	2.051667252	miR-27b-5p
CRELD1	4.122	2.043233258	miR-485-3p
ADTRP	4.083	2.029580189	miR-370-5p
MIA2	4.018	2.006443339	miR-543
ATP2B1	3.939	1.97800901	miR-370-5p
GREB1	3.825	1.935446116	miR-370-5p
ALG12	3.745	1.904838732	miR-27b-5p
HES6	3.702	1.888357965	miR-370-5p
STAC	3.701	1.887899472	miR-485-3p
OSGIN1	3.689	1.883395528	miR-485-3p
CCDC171	3.679	1.879368062	miR-543
SLFN13	3.546	1.826328727	miR-27b-5p
TMEM50B	3.476	1.797453063	miR-485-3p
ARHGAP25	3.447	1.785454243	miR-485-3p
SLC66A1	3.371	1.753302249	miR-485-3p
IRF1	3.343	1.741100497	miR-485-3p
PLEKHF1	3.322	1.732022665	miR-335-3p
CHKA	3.301	1.722922855	miR-485-3p
BRPF3	3.2	1.678275301	miR-370-5p
BHLHE40	3.195	1.675786665	miR-543
XAF1	3.17	1.664416108	miR-27b-5p
RAD9A	3.147	1.654166279	miR-27b-5p
GFPT1	3.137	1.649534168	miR-543
MFSD2A	3.098	1.631212935	miR-543
CLCN3	3.055	1.61129876	miR-370-5p
CPEB4	3.043	1.605695714	miR-543
GDPD1	3.032	1.600386761	miR-27b-5p
CLN3	3.021	1.594918398	miR-370-5p
PDE4D	2.982	1.576332418	miR-370-5p
PCSK9	2.967	1.568852352	miR-370-5p
FAM222A	2.959	1.565143101	miR-543
HM13	2.944	1.55791913	miR-370-5p
ZNRF2	2.895	1.533354778	miR-543
RRAGC	2.89	1.530936318	miR-543
PPP1R3F	2.878	1.525248408	miR-370-5p
NPTXR	2.862	1.516780852	miR-485-3p
ZFAND2A	2.852	1.512017251	miR-485-3p
TENT5A	2.802	1.486328385	miR-335-3p
SRGAP2B	2.742	1.455411827	miR-27b-5p
ARHGAP9	2.732	1.449779071	miR-370-5p
CFAP20DC	2.715	1.441083055	miR-485-3p
ST3GAL6	2.687	1.425949185	miR-370-5p
LRRC73	2.679	1.421550407	miR-335-3p

EPM2A	2.677	1.420368314	miR-370-5p
WASHC2A	2.652	1.406928969	miR-485-3p
GNS	2.651	1.40677278	miR-370-5p
TG	2.627	1.393144976	miR-485-3p
KCNA3	2.625	1.392356585	miR-543
POR	2.595	1.375489675	miR-27b-5p
CLCN6	2.548	1.349187013	miR-543
GID4	2.545	1.347649064	miR-543
PIP4P2	2.544	1.346992857	miR-485-3p
C19orf54	2.54	1.344919854	miR-370-5p
FBLL1	2.535	1.342075525	miR-27b-5p
KDSR	2.503	1.323788374	miR-370-5p
ABHD6	2.476	1.308119767	miR-485-3p
RNF13	2.443	1.288896218	miR-543
GOT1	2.437	1.284999561	miR-485-3p
LRRC1	2.398	1.261912125	miR-485-3p
MAP1LC3B	2.398	1.261823485	miR-370-5p
SLC25A37	2.366	1.242665474	miR-485-3p
UAP1L1	2.343	1.228425604	miR-370-5p
SELENOK	2.34	1.226705367	miR-370-5p
CBLB	2.338	1.225494324	miR-27b-5p
SOAT1	2.32	1.214242603	miR-370-5p
TTLL7	2.309	1.207118038	miR-543
ATP6V1B2	2.296	1.199094452	miR-370-5p
SOCS7	2.293	1.197056362	miR-370-5p
CERT1	2.287	1.193192588	miR-485-3p
FAM83G	2.266	1.180032393	miR-485-3p
AP3D1	2.224	1.152915003	miR-370-5p
SRGAP2C	2.223	1.152375328	miR-27b-5p
ELAPOR1	2.212	1.145540367	miR-485-3p
SLC31A1	2.212	1.145229711	miR-543
PNPLA3	2.2	1.137588721	miR-370-5p
ANKRD10	2.198	1.136190264	miR-370-5p
CPSF4	2.193	1.133157663	miR-485-3p
ZCCHC2	2.172	1.119256833	miR-485-3p
ASB1	2.147	1.102564742	miR-370-5p
VEGFB	2.144	1.100153816	miR-335-3p
HECA	2.141	1.098301213	miR-543
SYNGAP1	2.132	1.092218972	miR-370-5p
YIPF4	2.129	1.090066095	miR-543
BMF	2.121	1.084704082	miR-485-3p
ATF2	2.121	1.084514302	miR-27b-5p
KRT18	2.113	1.079591078	miR-27b-5p
MXI1	2.108	1.075587679	miR-543

FAM91A1	2.107	1.075312581	miR-27b-5p
LRP8	2.107	1.074994517	miR-27b-5p
OAS3	2.106	1.074550829	miR-485-3p
OSBPL3	2.096	1.067381897	miR-543
PPTC7	2.09	1.063580978	miR-543
EPHA2	2.085	1.059999106	miR-485-3p
RBCK1	2.073	1.051377918	miR-485-3p
ZFYVE27	2.067	1.047516363	miR-370-5p
KCTD13	2.054	1.038351419	miR-370-5p
PREB	2.05	1.035643151	miR-370-5p
TRPM7	2.04	1.028594435	miR-543
SLC1A3	2.023	1.016346712	miR-485-3p
MFSD9	2.015	1.010471808	miR-335-3p
RHOQ	2.01	1.006949722	miR-543
CTSA	2.004	1.002820123	miR-370-5p
SRI	0.497	-1.007362724	miR-194-5p
NME1	0.497	-1.008001514	miR-146a-5p
TEDC2	0.491	-1.027125399	miR-192-5p
ZNF106	0.489	-1.032774422	miR-192-5p
RNF141	0.489	-1.033546578	miR-29b-3p
CFL1	0.488	-1.036372847	miR-29b-3p
PEX19	0.487	-1.036687718	miR-29b-3p
DECR2	0.487	-1.038676146	miR-12136
RBX1	0.483	-1.048910686	miR-194-5p
RANBP6	0.481	-1.056423885	miR-194-5p
GYG1	0.48	-1.058972235	miR-194-5p
RASA4	0.479	-1.06052844	miR-194-5p
TM4SF1	0.479	-1.061367606	miR-148a-3p
UACA	0.478	-1.064953077	miR-29b-3p
CHN1	0.477	-1.067434786	miR-194-5p
PRMT7	0.475	-1.075234116	miR-137-3p
ALDH7A1	0.473	-1.080473249	miR-146a-5p
PXYLP1	0.472	-1.082127911	miR-29b-3p
ARF5	0.471	-1.086838556	miR-29b-3p
SOX6	0.471	-1.086915326	miR-194-5p
BPHL	0.469	-1.093691722	miR-194-5p
SEPTIN6	0.467	-1.097458904	miR-148a-3p
RFC2	0.466	-1.101408075	miR-146a-5p
ARHGAP20	0.463	-1.111957743	miR-148a-3p
TSPAN9	0.46	-1.120461687	miR-29b-3p
LAMA4	0.454	-1.140245434	miR-148a-3p
SEPTIN11	0.452	-1.144905881	miR-194-5p
TMEM237	0.452	-1.145092089	miR-29b-3p
FBXO6	0.45	-1.153333028	miR-192-5p

TMEM256-PLSCR3	0.448	-1.158449504	miR-29b-3p
TRPV4	0.446	-1.164329772	miR-29b-3p
SRSF2	0.445	-1.168261785	miR-148a-3p
EEF1AKMT2	0.444	-1.169862612	miR-148a-3p
ATP2B4	0.44	-1.183947492	miR-29b-3p
ABRAXAS1	0.439	-1.186083686	miR-194-5p
FGD6	0.435	-1.201520602	miR-194-5p
COL4A5	0.434	-1.205585085	miR-29b-3p
ABCC6	0.433	-1.208165472	miR-146a-5p
C10orf143	0.432	-1.210229622	miR-146a-5p
SUB1	0.431	-1.213608273	miR-148a-3p
MMP11	0.431	-1.215463726	miR-29b-3p
CARD10	0.428	-1.223901777	miR-146a-5p
PTPRD	0.427	-1.228280646	miR-194-5p
ROR1	0.426	-1.230051287	miR-29b-3p
SDC4	0.424	-1.236215855	miR-194-5p
AK4	0.423	-1.240589265	miR-148a-3p
CCDC152	0.422	-1.244877164	miR-192-5p
C1orf21	0.421	-1.247023163	miR-194-5p
GLIPR2	0.421	-1.249281766	miR-148a-3p
RASA4B	0.42	-1.251390024	miR-194-5p
NF2	0.42	-1.251747264	miR-146a-5p
HHIP	0.419	-1.256680089	miR-194-5p
HSPG2	0.407	-1.295266574	miR-146a-5p
ANGPTL4	0.407	-1.297275941	miR-194-5p
CACNB3	0.407	-1.29842612	miR-192-5p
CHML	0.406	-1.301368705	miR-146a-5p
MYLK	0.401	-1.316884043	miR-192-5p
CAV2	0.399	-1.325637128	miR-12136
CREB5	0.39	-1.359959653	miR-192-5p
ENPP1	0.389	-1.360999906	miR-194-5p
PKNOX2	0.387	-1.367907244	miR-29b-3p
GSN	0.386	-1.371789146	miR-148a-3p
PRPS1	0.384	-1.381516233	miR-146a-5p
PXMP2	0.383	-1.385620305	miR-29b-3p
LIMCH1	0.382	-1.387643114	miR-194-5p
PET100	0.38	-1.397423533	miR-192-5p
PLSCR3	0.379	-1.400721556	miR-29b-3p
AOPEP	0.373	-1.42176815	miR-146a-5p
SCRN2	0.363	-1.463355188	miR-29b-3p
LSAMP	0.362	-1.46460246	miR-194-5p
TNFAIP8	0.361	-1.470720256	miR-146a-5p
PXMP4	0.358	-1.480569977	miR-29b-3p

RALGPS2	0.355	-1.495836697	miR-194-5p
ANXA2	0.353	-1.501677206	miR-29b-3p
GSTM2	0.352	-1.505433741	miR-148a-3p
LRRC8B	0.348	-1.524872542	miR-148a-3p
FAM229B	0.345	-1.535357378	miR-192-5p
ACTA2	0.344	-1.540984468	miR-146a-5p
PIGB	0.337	-1.570542418	miR-146a-5p
A2M	0.334	-1.581351915	miR-146a-5p
ADGRL2	0.329	-1.602529685	miR-194-5p
IFITM1	0.321	-1.641292811	miR-194-5p
STMN1	0.316	-1.662204236	miR-194-5p
DDAH1	0.316	-1.663520878	miR-29b-3p
WNK4	0.31	-1.688213927	miR-146a-5p
CCDC88B	0.303	-1.722517024	miR-146a-5p
SOX11	0.299	-1.741024154	miR-194-5p
PCSK5	0.294	-1.76800081	miR-29b-3p
COL5A1	0.29	-1.783842698	miR-192-5p
C5orf34	0.287	-1.80219237	miR-146a-5p
BARD1	0.284	-1.817159332	miR-192-5p
PDGFD	0.261	-1.940245187	miR-29b-3p
SETBP1	0.26	-1.945307346	miR-194-5p
SERPINB9	0.256	-1.963583407	miR-29b-3p
MTARC1	0.255	-1.972703899	miR-194-5p
CAT	0.254	-1.97950594	miR-146a-5p
FANCA	0.252	-1.990421226	miR-194-5p
MEX3A	0.25	-1.999434592	miR-194-5p
MEST	0.243	-2.043128757	miR-29b-3p
FERMT1	0.237	-2.079104393	miR-146a-5p
ACADS	0.235	-2.090711433	miR-192-5p
DEPDC1B	0.235	-2.091185655	miR-194-5p
ARNT2	0.233	-2.1019904	miR-148a-3p
KIAA1549L	0.233	-2.104450933	miR-194-5p
GARNL3	0.232	-2.109881754	miR-146a-5p
GINS4	0.232	-2.109981683	miR-192-5p
STXBP6	0.222	-2.171396329	miR-148a-3p
SCUBE3	0.221	-2.181065684	miR-29b-3p
PDE5A	0.219	-2.188645908	miR-137-3p
SLC7A14	0.21	-2.24992727	miR-148a-3p
KCND2	0.201	-2.311776255	miR-194-5p
AC087632.2	0.201	-2.317767904	miR-146a-5p
AQP3	0.194	-2.368690539	miR-29b-3p
NEDD9	0.183	-2.448428747	miR-146a-5p
NDRG2	0.17	-2.553712458	miR-148a-3p
CCNA2	0.163	-2.613705527	miR-29b-3p

VASH2	0.153	-2.706828732	miR-29b-3p
OIP5	0.148	-2.753896368	miR-194-5p
CSRNP3	0.147	-2.765900477	miR-146a-5p
PMP22	0.146	-2.777561276	miR-29b-3p
FNDC5	0.133	-2.914170205	miR-29b-3p
OLFM1	0.129	-2.950608913	miR-148a-3p
NREP	0.123	-3.025819063	miR-137-3p
MCM10	0.122	-3.034446067	miR-194-5p
DKKL1	0.11	-3.187875375	miR-146a-5p
AURKB	0.109	-3.196646781	miR-148a-3p
HMMR	0.101	-3.305753915	miR-194-5p
ACTN3	0.098	-3.352952197	miR-29b-3p
THBS1	0.097	-3.358613587	miR-194-5p
CDKN3	0.086	-3.53352172	miR-146a-5p
GUCY1B1	0.085	-3.556282931	miR-146a-5p
RAD54L	0.081	-3.631623471	miR-146a-5p
KCNJ6	0.078	-3.684577323	miR-192-5p
LBH	0.069	-3.85861911	miR-192-5p
PTGIS	0.063	-3.996623614	miR-146a-5p
PRC1	0.062	-4.008707055	miR-148a-3p
TMEM176A	0.06	-4.057066489	miR-29b-3p
JPH2	0.046	-4.455913027	miR-146a-5p
ELN	0.043	-4.552607873	miR-29b-3p
PDE7B	0.04	-4.636946512	miR-29b-3p
RAB7B	0.032	-4.959717889	miR-29b-3p
