# Supplementary material for: High Stretch Modulates cAMP/ATP Level in Association with Purine Metabolism via miRNA–mRNA Interactions in Cultured Human Airway Smooth Muscle Cells
Source: Cells. 2024 Jan 5;13(2):110. doi: 10.3390/cells13020110 (PMC10813996; doi:10.3390/cells13020110)
Supplement: Supplementary file 1 [file cells-13-00110-s001.zip › Table S3 KEGG pathway.pdf]

Table S3. Significant enriched KEGG pathway of 283 target DE-mRNAs.

KEGG ID	KEGG term	Count	P_{Value}	Genes
hsa00230	Purine metabolism	9	0.001	PRPS1, GUCY1B1, PDE4D, ENPP1, AK4, PDE5A, PDE7B, AK7, NME1
hsa04512	ECM-receptor interaction	7	0.015	SDC4, COL5A1, LAMA4, COL4A5, HMMR, THBS1, HSPG2
hsa03440:	Homologous recombination	4	0.029	BARD1, ABRAXAS1, RAD54L, BRCA2
hsa04146	Peroxisome	5	0.046	PEX19, PXMP2, CAT, PXMP4, DECR2
hsa04510	Focal adhesion	10	0.051	ACTN3, COL5A1, CAV2, LAMA4, PDGFD, KDR, VEGFB, COL4A5, THBS1, MYLK
hsa04022	cGMP-PKG signaling pathway	7	0.058	ATF2, GUCY1B1, ATP2B4, PDE5A, ATP2B1, CREB5, MYLK
hsa04010	MAPK signaling pathway	10	0.062	IL1A, ATF2, CACNB3, PTPRR, PDGFD, STMN1, MKNK2, KDR, VEGFB, EPHA2
hsa04978	Mineral absorption	4	0.076	SLC31A1, ATP2B4, TRPM7, ATP2B1
hsa05418	Fluid shear stress and atherosclerosis	6	0.080	IL1A, GSTM2, SDC4, CAV2, TRPV4, KDR
hsa05146	Amoebiasis	7	0.088	ACTN3, COL5A1, LAMA4, COL4A5, SERPINB9, RAB7B, TLR2
hsa04014	Ras signaling pathway	8	0.096	RASA4B, SYNGAP1, RASA4, PDGFD, PLCG2, KDR, VEGFB, EPHA2
