# Supplementary material for: High Stretch Modulates cAMP/ATP Level in Association with Purine Metabolism via miRNA–mRNA Interactions in Cultured Human Airway Smooth Muscle Cells
Source: Cells. 2024 Jan 5;13(2):110. doi: 10.3390/cells13020110 (PMC10813996; doi:10.3390/cells13020110)
Supplement: Supplementary file 1 [file cells-13-00110-s001.zip › Table S4 GO enrichment of target DE-mRNAs in ASMCs.pdf]

Table S4. Significant enrichment of GO analysis of target DE-mRNAs in ASMCs.

GO ID	GO term	Category	Count	P_{Value}	Genes
GO:0006816	Calcium ion transport	BP	7	0.001251	EPM2A, CACNB3, TRPV4, TRPM7, SLC3A2, SRI, SELENOK
GO:0043087	Regulation of GTPase activity	BP	7	0.001404	ARHGAP9, FGD6, RASA4B, SYNGAP1, RASA4, CHN1, CBLB
GO:0007165	signal transduction	BP	31	0.002096	ARHGAP9, PTPRR, HHIP, WNK4, NEDD9, CBLB, SRI, NDRG2, LRP8, SCUBE3, FAM83G, ARHGAP20, SYNGAP1, STMN1, CHN1, LRRC8B, CERT1, STAC, PDE4D, OSGIN1, ARHGAP25, FNDC5, OLFM1, CLCN6, TG, RASA4, PDE5A, PDE7B, RHOQ, F2RL3, TLR2
GO:0071711	Basement membrane organization	BP	4	0.003029	MMP11, FERMT1, FLRT2, CAV2
GO:0009267	Cellular response to starvation	BP	6	0.003338	MAP1LC3B, RRAGC, RRAGD, PCSK9, MFSD2A, ELAPOR1
GO:0045766	Positive regulation of angiogenesis	BP	8	0.006696	IL1A, PTGIS, DDAH1, VASH2, KDR, VEGFB, ANGPTL4, THBS1
GO:0046034	ATP metabolic process	BP	4	0.009631	ABCC6, ATP6V1B2, ENPP1, AK4
GO:0055074	Calcium ion homeostasis	BP	4	0.010461	JPH2, ABCC6, WNK4, KDR
GO:0016477	Cell migration	BP	10	0.01209	CLN3, DEPDC1B, SDC4, COL5A1, KCTD13, KDR, NEDD9, THBS1, RHOQ, EPHA2
GO:0032755	Positive regulation	BP	6	0.012537	IL1A, TRPV4, PLCG2, SELENOK, RAB7B, TLR2

	of interleukin-6 production				
GO:0030336	Negative regulation of cell migration	BP	8	0.012587	SRGAP2C, IFITM1, MCTP1, LIMCH1, NEDD9, ABHD6, NF2, SRGAP2B
GO:0032760	Positive regulation of tumor necrosis factor production	BP	6	0.014056	IL1A, OAS3, PLCG2, SELENOK, THBS1, TLR2
GO:0097284	Hepatocyte apoptotic process	BP	3	0.014622	ATF2, KRT18, GSN
GO:0055119	Relaxation of cardiac muscle	BP	3	0.014622	GSTM2, GSN, PDE5A
GO:0071639 production	Positive regulation of monocyte chemotactic protein-1	BP	3	0.016724	OAS3, TRPV4, SELENOK
GO:0051480	Regulation of cytosolic calcium ion concentration	BP	4	0.018562	CLN3, CAV2, ATP2B4, ATP2B1
GO:0030513	Positive regulation of BMP signaling pathway	BP	4	0.018562	SCUBE3, KDR, KCP, SOX11
GO:0035556	Intracellular signal transduction	BP	13	0.021471	GUCY1B1, RASA4B, STAC, WNK4, CBLB, DEPDC1B, RASA4, CHN1, STMN1, MKNK2, PLCG2, SOCS7, ASB1
GO:0001666	Response to hypoxia	BP	7	0.021519	IL1A, ARNT2, CAT, VEGFB, ANGPTL4, THBS1, TLR2
GO:0044090	Positive regulation	BP	2	0.026606	ANXA2, ELAPOR1

	of vacuole organization				
GO:0005515	Protein binding	MF	200	0.00189884	ATF2, IFITM1, HHIP, CBLB, IFI30, AQP3, MYLK, ZFYVE27, SYNGAP1, STMN1, KDR, FBXO6, SOX6, EPHB1, PKNOX2, HES6, ACTN3, BHLHA15, HSPG2, TM4SF1, POR, RRAGC, SOAT1, LRATD2, SUB1, RRAGD, KCTD13, PREB, RBCK1, ALDH7A1, ATF3, EPHA2, SDC4, MTARC1, WNK4, GNS, PRDM16, PLCG2, A2M, DECR2, BARD1, GSTM2, STAC, TMEM50B, RAB3IP, OSGIN1, CCDC88B, OCLN, TG, TMCC3, HYOU1, CPEB4, HRK, ARHGAP9, PTPRR, YJEFN3, ZFAND2A, ELN, SLC7A14, MIA2, PCSK9, BRCA2, PCSK5, MAP1LC3B, PIP4P2, JPH2, RALGPS2, PRMT7, KCND2, ANXA2, SERPINB9, RENBP, NME1, IL1A, PLSCR3, CRELD1, ANGPTL4, RAB7B, ARF5, RHOQ, TLR2, KCNA3, NEDD9, HSPA13, CHN1, RAD54L, ARNT2, PEX19, GSN, SLC31A1, YIPF4, TMEM176A, GINS4, AP3D1, VEGFB, LSAMP, SEPTIN6, C1ORF21, RANBP6, KRT18, RNF141, CDKN3, GUCY1B1, LRRC73, WASHC2A, TGM1, CFL1, SLC16A6, ENPP1, OIP5, GYG1, EPM2A, CARD10, PPTC7, VASH2, FAM91A1, RBX1, OLFM1, CACNB3, SRSF2, ROR1, ADTRP, CCDC152, TEDC2, SEL1L, SLC1A3, ABHD6, AK4, HMMR, NREP, NDRG2, FAM83G, SCUBE3, ANKRD10, BMF, KDSR, SOCS7, CERT1, CPSF4, BRPF3, CAV2, OSBPL3, FANCA, FAM229B, PTPRD, COL5A1,

					TMEM237, TRPV4, PXMP2, BHLHE40, PXMP4, NF2, XAF1, RAD9A, TNFAIP8, PLEKHF1, RNF13, MCM10, DMKN, SEPTIN11, LBH, SCRN2, PTGIS, RFC2, PDE4D, TENT5A, KLF15, CCNA2, SETBP1, IRF1, OAS3, ATP6V1B2, PXYLP1, PDE5A, CREB5, PRPS1, ABRAXAS1, HM13, SLC3A2, SRI, SELENOK, CLCN3, LRP8, UACA, THBS1, AURKB, CLN3, SPOCK2, MKNK2, MXI1, REPS2, LRRC8B, MEST, KCNJ6, ATP2B4, ATP2B1, SLC25A37, PRC1, PMP22, ASB1
GO:0042802	Identical protein binding	MF	39	0.002739707	ATF2, PRPS1, SDC4, MCM10, SRI, SELENOK, BRCA2, THBS1, AQP3, TGM1, SCUBE3, ZFYVE27, KDR, OIP5, A2M, CERT1, ACTN3, CHKA, ANXA2, SLC31A1, RAB3IP, VEGFB, BHLHA15, RENBP, NME1, TG, SOAT1, PRC1, TRPV4, SUB1, CAT, KCTD13, TMCC3, RBCK1, ANGPTL4, ALDH7A1, ATF3, CREB5, TLR2
GO:0002020	Protease binding	MF	7	0.004716294	ANXA2, SERPINB9, SRI, A2M, BRCA2, THBS1, F2RL3
GO:0005096	GTPase activator activity	MF	11	0.007735201	ARHGAP9, ARHGAP20, DEPDC1B, RASA4B, SYNGAP1, RASA4, CHN1, PREB, CHML, ARHGAP25, GARNL3
GO:0048306	Calcium-dependent protein binding	MF	6	0.009035789	CLN3, PLSCR3, ANXA2, ATP2B4, A2M, LRP8
GO:0051020	GTPase binding	MF	4	0.012732134	RRAGC, RRAGD, RAB3IP, PREB
GO:0097110	scaffold protein binding	MF	5	0.014410881	KRT18, CAV2, PDE4D, PLCG2, ATP2B4
GO:0004115	3',5'-cyclic-AMP phosphodiesterase activity	MF	3	0.020556356	PDE4D, ENPP1, PDE7B

GO:0005178	Integrin binding	MF	7	0.023901888	FERMT1, ACTN3, COL5A1, KDR, NF2, THBS1, HSPG2
GO:0098641	Cadherin binding involved in cell-cell adhesion	MF	3	0.025734396	KRT18, ANXA2, STXBP6
GO:0005524	ATP binding	MF	32	0.026270808	PRPS1, WNK4, AK4, CLCN3, AK7, HSPA13, AURKB, MYLK, MKNK2, KDR, ENPP1, RAD54L, TRPM7, CNNM2, EPHB1, CHKA, RFC2, ABCC6, ATP2B4, ATP2B1, RENBP, NME1, TTLL7, ACTA2, CLCN6, SLFN13, OAS3, TRPV4, ATP6V1B2, HYOU1, ROR1, EPHA2
GO:0042803	Protein homodimerization activity	MF	18	0.030217761	BARD1, EPM2A, ATF2, PRPS1, GSTM2, CHKA, CAV2, HM13, THBS1, SRGAP2C, GLIPR2, KYNU, BHLHE40, CAT, ENPP1, SOX6, GYG1, ATF3
GO:0004550	Nucleoside diphosphate kinase activity	MF	3	0.031379982	AK4, AK7, NME1
GO:0017124	SH3 domain binding	MF	6	0.032164615	SYNGAP1, PLSCR3, CBLB, ZNF106, SOCS7, RAD9A
GO:0004114	3',5'-cyclic-nucleotide phosphodiesterase activity	MF	3	0.034368752	PDE4D, PDE5A, PDE7B
GO:0004714	Transmembrane receptor protein tyrosine kinase activity	MF	4	0.037972433	KDR, ROR1, EPHB1, EPHA2

GO:0038024	Cargo receptor activity	MF	3	0.040660243	MIA2, HMMR, LRP8
GO:0016301	Kinase activity	MF	8	0.04263756	PRPS1, CHKA, MKNK2, AK7, CERT1, EPHA2, CDKN3, MYLK
GO:0046872	Metal ion binding	MF	49	0.043376334	ATF2, GUCY1B1, PLEKHF1, RNF13, MCM10, MYLK, TGM1, ZFYVE27, ENPP1, OIP5, RASA4B, KCND2, PPTC7, LIMCH1, PDE4D, KLF15, DDAH1, RASA4, OAS3, CAT, PDE5A, RBCK1, NPTXR, CREB5, GDPD1, SLC1A3, GNS, AURKB, ZNRF2, FGD6, MKNK2, CHN1, PRDM16, RAD54L, ZNF106, TRPM7, BARD1, CPSF4, BRPF3, STAC, ATP2B4, ATP2B1, TTLL7, MEX3A, COL5A1, TRPV4, PDE7B, RNF141, CPEB4
GO:0003824	Catalytic activity	MF	6	0.04473424	DDAH1, MTARC1, KYNU, HHIP, SLC3A2, MEST
GO:0005201	Extracellular matrix structural constituent	MF	6	0.04473424	COL5A1, LAMA4, ELN, COL4A5, CRELD1, THBS1

BP: biological process; CC: cellular component; MF: molecular function.
