# Supplementary material for: High Stretch Modulates cAMP/ATP Level in Association with Purine Metabolism via miRNA–mRNA Interactions in Cultured Human Airway Smooth Muscle Cells
Source: Cells. 2024 Jan 5;13(2):110. doi: 10.3390/cells13020110 (PMC10813996; doi:10.3390/cells13020110)
Supplement: Supplementary file 1 [file cells-13-00110-s001.zip › Table S5 KEGG enrichment of first module of PPI.pdf]

Table S5. Significant enrichment of KEGG of first module of PPI.

KEGG ID	KEGG term	Count	P_{Value}	Genes
hsa00230	Purine metabolism	7	3.64E-06	PDE4D, ENPP1, AK4, PDE5A, PDE7B, AK7, NME1
hsa01232	Nucleotide metabolism	4	0.002916	ENPP1, AK4, AK7, NME1
hsa03440	Homologous recombination	3	0.008482	BARD1, RAD54L, BRCA2
hsa00380	Tryptophan metabolism	3	0.008888	KYNU, CAT, ALDH7A1
hsa01100	Metabolic pathways	12	0.009186	PDE4D, KYNU, CAT, ENPP1, AK4, PDE5A, PDE7B, GYG1, AK7, ACADS, ALDH7A1, NME1
hsa01240	Biosynthesis of cofactors	4	0.014804	KYNU, AK4, AK7, NME1
