# Supplementary figures and images for: High Stretch Modulates cAMP/ATP Level in Association with Purine Metabolism via miRNA–mRNA Interactions in Cultured Human Airway Smooth Muscle Cells
Source: Cells. 2024 Jan 5;13(2):110. doi: 10.3390/cells13020110 (PMC10813996; doi:10.3390/cells13020110)

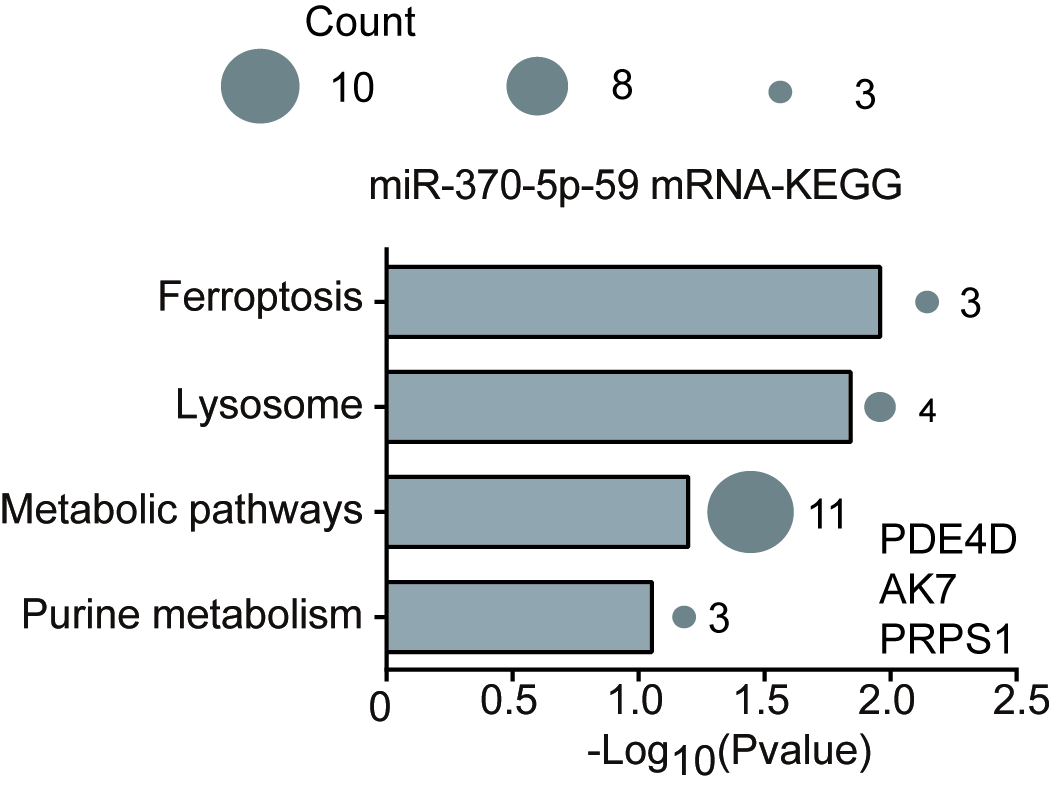

Supplement: Supplementary file 1 [file cells-13-00110-s001.zip › Figure S1 miR-370 mRNA-KEGG.tif]

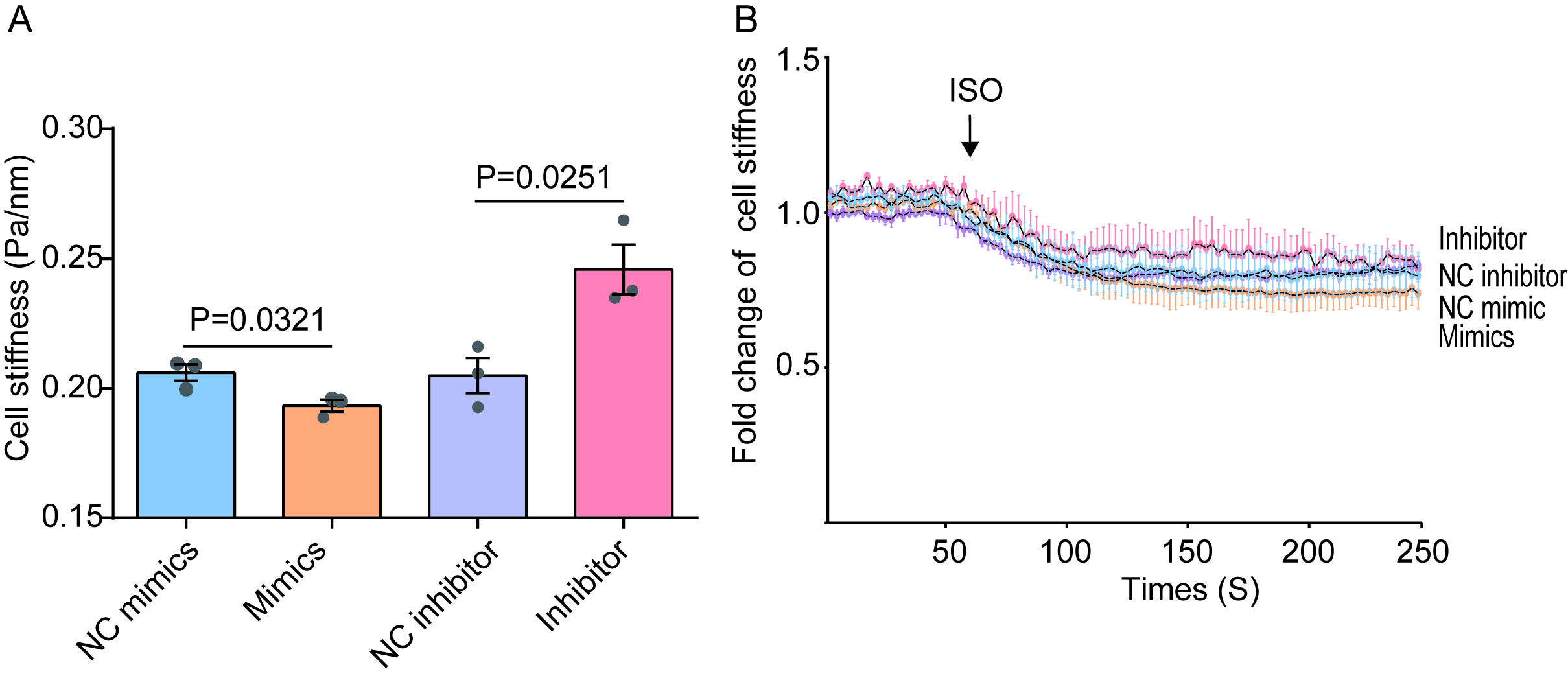

Supplement: Supplementary file 1 [file cells-13-00110-s001.zip › Figure S2 new.tif]
